# Urban Expansion Was the Main Driving Force for the Decline in Ecosystem Services in Hainan Island during 1980–2015

**DOI:** 10.3390/ijerph192315665

**Published:** 2022-11-25

**Authors:** Jia Geng, Mingsheng Yuan, Shen Xu, Tingting Bai, Yang Xiao, Xiaopeng Li, Dong Xu

**Affiliations:** 1 International Hospitality Management School, University of Sanya, Sanya 572000, China; 2 School of Business Administration, Northeastern University, Shenyang 110819, China; 3 School of Psychological and Cognitive Sciences, Peking University, Beijing 100871, China; 4 Academician Workstation of Zhai Mingguo, University of Sanya, Sanya 572022, China; 5 The Third Engineering Co., Ltd. of China Railway 22nd Bureau Group, Xiamen 361000, China; 6 State Key Laboratory of Remote Sensing Science, Beijing Normal University, Beijing 100091, China

**Keywords:** Hainan Island, land use change, ecosystem services, spatiotemporal change, driving forces

## Abstract

Hainan Island is one of China’s most ecologically diverse areas. Human activities and climate change have recently influenced Hainan Island’s ecosystem services. Therefore, scientific methods are urgently needed to investigate the characteristics of these services’ spatial and temporal variations and their driving mechanisms for maintaining Hainan Island’s biodiversity and high-quality ecological conservation. Based on multivariate remote sensing and reanalysis data, this study analysed the spatial and temporal variations in water retention, soil conservation, carbon sequestration, and oxygen release services on Hainan Island during 1980–2015 using various ecosystem service models such as INVEST, CASA and RULSE. Then, we analysed different ecosystem service drivers using a random forest model. The results indicated that (1) from 1980 to 2015, the change characteristics of different ecosystem types (arable, forest, and grassland) decreased, and the proportion of decrease was 0.98%, 0.55% and 0.36%, respectively. Built-up and water increased significantly, and the proportion of increase reached 1.46% and 0.51%, respectively. (2) Hainan Island’s functions of water retention, soil conservation, carbon sequestration, and oxygen release services decreased from 23.31 billion m3, 2.89 billion t, 9.68 million t and 56.05 million t in 1980 to 23.15 billion m3, 2.79 billion t, 9.42 million t and 55.53 million t in 2015, respectively. The high value area was mainly distributed in Hainan Island’s central mountainous area, and the low value area was mainly distributed in the lower-elevation coastal area. (3) In the past 35 years, urban expansion has been the leading factor in the reduction of Hainan Island’s ecosystem service capacity. However, its central nature reserve and other forms of ecological protection have improved its ecosystem service capacity, which has alleviated the overall declining trend of its amount of ecosystem service functions. (4) The driving forces for the spatial distribution of Hainan Island’s ecosystem services were analysed using a random forest algorithm, which indicated that its spatial distribution was mainly driven by rainfall, soil moisture, actual evapotranspiration, maximum temperature, and minimum temperature. This study is expected to help planners develop effective environmental policies to accommodate the potential ecological risks associated with urban expansion during the construction of Hainan Island’s future free trade port while filling the gaps in existing studies.

## 1. Introduction

As an important refuge for natural resources and biodiversity, it has one of the highest eco-environmental qualities in China. Hainan Island’s ecosystem is important for maintaining the regional ecological balance as well as protecting China’s and the world’s biodiversity [1]. However, in recent decades, with increasing human activities caused by increased regional economic and political attention in the South China Sea, the structure and function of the Hainan Island ecosystem have undergone drastic changes [2,3,4]. In particular, in the last 20 years, rapid urbanization and increasing population density have jointly driven accelerated changes in the urban substrate of Hainan Island, affecting ecosystem services and eco-efficiency [5,6,7].

Moreover, the current and future economic development of Hainan needs a deep understanding of its ecosystem advantages and limitations. Hainan was designated as one of China’s first special economic zones in 1988 [8]. However, it was relatively underdeveloped compared to other regions in China. Hainan’s economy only had a small industrial sector and depended heavily on service sectors and nature tourism resources. In April 2018, China announced plans to establish the Hainan Free Trade Zone by 2020 and build Hainan Free Trade Port by 2025 [9]. Hainan will become a strategic trade and investment destination in China. When new economic opportunities are created in Hainan, human activities will depend on and affect Hainan’s ecosystem. High quality ecosystem services can play a positive role in attracting investment and promoting development. However, compared to terrestrial ecosystems, tropical island ecosystems are relatively homogeneous in structure, and buffer capacity is weak [10]. Tropical island ecosystems are vulnerable to invasive alien species and anthropogenic disturbances, in particular, frequent disturbances such as construction and industrial production are likely to cause fragmentation of natural habitats on Hainan Island, which will directly lead to a decrease in habitat pattern connectivity and an increase in spatial heterogeneity. With the future construction and development of Hainan Island, the integration of tropical island ecosystems in line with the characteristics of low disturbance and an ecologically sustainable economic development model of tropical islands is imperative. Therefore, revealing the spatial and temporal evolution characteristics of ecosystem services on Hainan Island and their driving mechanisms is of great practical significance for ecological protection and the realization of sustainable development goals during the future construction of the Hainan Free Trade Port.

As a ‘bridge’ between human society and natural ecosystems, ecosystem services convey natural ecosystems’ well-being to human society [11,12], i.e., the natural environmental benefits that ecological processes and ecosystems create and sustain and that is the environmental and resource basis for human survival and development [13]. Ecosystem service functions include supply, regulation, support, and cultural service functions that interact with natural resources at the spatial scale [14], thus reflecting direct and indirect as well as tangible and intangible benefits that humans obtain [15,16]. Determining how to reconcile human activities with ecological patterns and resource environments is the core issue of human-earth relations [17]. As a result, ecosystem service research emphasizes ecosystems’ structure, function, process, and sustainable resource supply capacity [18] to discern the characteristics of dominant ecosystem services as well as the spatial and temporal distribution in different regions and to provide scientific support for regional ecological management [19]. From an integrated thinking perspective, ecosystem services can be a way to allocate resources and coordinate interests and are an important tool to spatially optimize national territory [20] as well as to promote planning decisions aligned with the concept of ecological civilization [21]. In recent years, the rapid development of Earth observation systems has greatly promoted the study of ecosystem services in large-scale regions [3,4], and these studies have improved the understanding of spatial ecosystem services. However, through combing and summarizing, this paper finds that these studies still have some limitations.

Due to the uniqueness and importance of Hainan Island’s ecosystem service functions [22], scholars have started early research on its ecosystem services, especially for the ecosystem service functions played by tropical rainforests [23] and mangrove ecosystems [24] in its central mountainous region. These studies also assessed Hainan Island’s overall ecosystem services and values [1], but the results are mostly based on single-year data. Fortunately, Lei et al. [25] conducted a long time series analysis of the value of Hainan Island’s ecosystem services and portrayed land use’s impact on the value of ecosystem services. However, since this analysis incorporated the value-equivalent factor method [26], it could not present the characteristics of the spatial and temporal dynamics of ecosystem services that are closely related to ecological structure and function [27]. In addition, most of the current studies only focus on the assessment of a single ecosystem service function on Hainan Island, and there is a lack of multifunctional assessment studies. Ang Li et al. [28] explored the effect of land use change on water production in Hainan Island Tropical Rainforest National Park from 1990–2018 using the Invest model. Lei Jinrui et al. [29] used the Invest model to reveal the spatial and temporal evolutionary characteristics of habitat quality in three major watersheds of Hainan Island from 1980 to 2020. However, a single function cannot comprehensively characterize the good or bad ecosystem service function of Hainan Island. Finally, for ecosystem service driver analysis, most existing studies have explored the temporal dimension. However, the lack of thinking in spatial dimensions hinders a broader understanding of the spatial distribution of ecosystem service functions.

Therefore, based on the ecological process model, this study combined a multisource remote sensing dataset and reanalysis data to explore the spatial and temporal variations in the four more important types of ecosystem services (water content, soil conservation, carbon sequestration services, and oxygen release services) on Hainan Island from 1980 to 2015 and explored the drivers of the spatial distribution of ecosystem services on Hainan Island over the last 35 years using the random forest [30] model. The response patterns of different ecosystem services were compared with human activities and natural elements over time. This evaluation of ecosystem services aims to provide a basis for the rational allocation and utilization of land resources, coordination of human activities, protection of ecological patterns, and optimization of ecosystem management during the construction of the Hainan Free Trade Port [31,32].

## 2. Materials and Methods

### 2.1. Study Area

**Figure 1:**
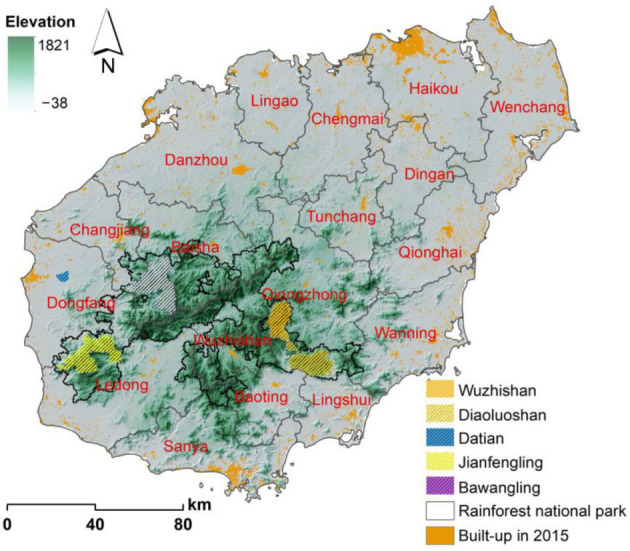
Map of Hainan Island.

Hainan Island (108.37°–111.03° E, 18.80°–20.10° N) is located at the northern end of the continental shelf in the South China Sea, facing Guangdong Province across the Qiongzhou Strait to the north and Guangxi and Vietnam across the Gulf of Tonkin to the west [33]. The study area has a tropical monsoon climate with its most important features of a long summer and no winter. It is known as a ‘natural greenhouse’ [34], with a multiyear average temperature of 22–27 °C and a multiyear average annual total rainfall fluctuating between 1000–2000 mm [35]. Hainan Island’s topography is low around its coastline and high in its middle, with Wuzhi Mountain and Parrot Mountain in the middle as the core of the rise and gradually decreasing in all directions. Most of Hainan Island’s mountains are between 500 and 800 m high and create a hilly and low terrain. Mountains, hills, terraces, and plains form a circular layered landform with a clear gradational structure (Figure 1). The establishment of the Hainan Tropical Rainforest National Park [36] in 2020, with a total area of 4400 km^2^ and accounting for one-seventh of Hainan Island’s land area, which is one of the 34 biodiversity hotspots in the world, is intended to maintain the originality, integrity, and diversity of its tropical rainforest ecosystem [37].

Hainan is surrounded by the sea, and its ecosystem has a weak ability to resist disturbance, and its independent and closed ecological environment makes it a sensitive area to climate change [38]. Second, in the process of accelerating the construction of a free trade port with Chinese characteristics and a national pilot ecological civilization zone, economic development and urban development have led to a shift in regional land use types and increased land fragmentation, especially in the last decade, accelerated urban expansion and rapid increase in construction land, which have profoundly affected island-wide ecosystem services [25,39].

### 2.2. Data Sources

The data used to estimate ecosystem services in this study mainly include China’s Land-Use/Cover Datasets (CLUD) [40], sunshine hour data, soil texture, soil organic matter content, soil capacity data, national nature reserve data, meteorological data, base pond area, inlet and outlet water data, ecosystem forest net productivity NPP data (calculated by the CASA model [41]), administrative division data, and digital elevation model data. Data for exploring the drivers of the spatial distribution of ecosystem services mainly include actual evapotranspiration (AET), water deficit (DEF), potential evapotranspiration (PET), surface runoff (RO), soil moisture (SOIL), shortwave downgradient radiation (SRAD), minimum temperature (TMN), maximum temperature (TMX), saturated water vapour pressure difference (VPD), ten-meter wind speed (VS), total annual precipitation (PRE), and normalized vegetation index (calculated from Landsat image [42]). The data used in this study are detailed in Table 1. The data preprocessing process in this study included a unified coordinate system (WGS 1984) with a spatial resolution of 30 m, as well as monthly data synthesized into annual data. All data preprocessing was performed in ArcGIS 10.8 [43]. In addition, it should be noted that the remote sensing data and reanalysis data used in this study were obtained from Google Earth Engine [44].

Hainan LULC data from 1980 to 2015 was obtained from the Resource and Environmental Science and Data Center (https://www.resdc.cn/, accessed on 5 May 2022). These data were produced at a resolution of 30 m, using visual interpretation of Landsat images, with an average overall accuracy greater than 94%. Meteorological data (including precipitation, temperature and total solar radiation on a monthly scale) from 1980 to 2015 was provided by the Chinese National Metrological Information Center of the China Meteorological Administration (http://data.cma.cn, accessed on 5 May 2022). Digital elevation model (DEM) data originated from the Shuttle Radar Topography Mission (SRTM), with a resolution of 30 m (https://lpdaac.usgs.gov/, accessed on 5 May 2022). The average annual potential evapotranspiration (ETo) was obtained by the modified Hargreaves equation. The soil map and associated soil attributes (including particle composition, nutrient content, soil depth and soil bulk density) were acquired from the Institute of Soil Science, Chinese Academy of Sciences. The watersheds and subwatersheds were generated by using ArcSWAT based on DEM data.

### 2.3. Methods

#### 2.3.1. Optimal Granularity of Ecosystem Type

Ecosystem types have obvious scale characteristics [46,47], and ecosystem-oriented solutions need appropriate scales [48]. Exploring the most sensitive spatial unit range of the ecological landscape index in response to changes in landscape components, namely, the optimal granularity [49], has an important scale indicator role for setting natural solutions oriented to ecosystem services. The coefficient of variation is a relative statistic used to measure the dispersion of serial observations [50,51]. This study used the coefficient of variation method to determine the sensitivity of each landscape index to the change in spatial granularity [52]. With the increase in landscape spatial granularity, if the coefficient of variation of a landscape index increases, it indicates that the landscape index is highly sensitive to spatial granularity, and the greater the coefficient of variation is, the higher the sensitivity, and vice versa. In this study, first, the vector data of landscape types in each year in the study area were rasterized in ArcGIS, and 28 landscape type data with different spatial granularities (30–300 m) were obtained in each year. Then, based on the study by Yutong Song et al. [53], at the landscape level, the representative landscape pattern index is selected from the four aspects of the area edge index, shape index, diversity index and aggregation index to calculate the coefficient of variation and select the sensitivity index. At the landscape level, the total landscape area (TA), number of patches (NP), patch density (PD), largest patch index (LPI), edge density (ED), landscape similarity index (LSI), average patch area (SHAPE-MN), perimeter area fractal dimension (PAFRAC), contagion index (CONTAG), the proportion of like adjacency (PLADJ), interspersion juxtaposition index (IJI), patch cohesion (COHESION), landscape division index (DIVISION), split index (SPLIT), Shannon diversity index (SHDI), Shannon evenness index (SHEI), and aggregation index (AI) are 17 landscape indexes. All landscape indexes were calculated in Fragstats software.

#### 2.3.2. Water Retention

The water supply function [54] is mainly expressed as the delayed function of increasing subsurface runoff, moderating surface runoff, and regulating river runoff by intercepting and retaining rainfall through the forest canopy, subsurface vegetation, deadfall layer, and soil layer. The water connotation function not only satisfies the water demand of the ecosystem components but also provides a certain amount of water for its downstream, which is in a key position among many ecological service functions [55]. In this paper, the water content is calculated by the water balance equation (WBE) [56].

(1) $$Q_{WC}=P_{i}-R_{i}-ET_{i}$$

where *Q_WC_* is water retention (mm yr^−1^); *P_i_* is rainfall (mm yr^−1^); *R_i_* is runoff (mm yr^−1^); and *ET_i_* is evapotranspiration (mm yr^−1^).

#### 2.3.3. Water Retention

The soil conservation function mainly refers to forest and grassland ecosystems’ function to reduce erosion energy from rainfall by gradually consuming each level of the forest canopy and deadfall layer, increasing soil erosion resistance and thereby reducing soil erosion and soil loss while maintaining soil [57]. The soil conservation function has an important position in ecosystem services, has a fundamental role in many aspects, such as soil formation, water containment, and vegetation fixation, and provides ecological security and system services. The principles of soil conservation calculations in this study are as follows [58,59].

Actual soil erosion:

(2) $$A_{AE}=R\times K\times L\times S\times C\times P$$

Potential soil erosion:

(3) $$A_{PE}=R\times K\times L\times S$$

Soil conservation:

(4) $$A_{SE}=A_{PE}-A_{AE}$$

where *A_AE_* is the actual soil erosion per unit area in (t hm^−2^ yr^−1^); *A_PE_* is the potential soil erosion per unit area in (t hm^−2^ yr^−1^); *A_SE_* is the soil retention per unit area in (t hm^−2^ yr^−1^); rainfall erosivity factor *R* represents the multiyear average annual rainfall erosivity index; soil erodibility factor *K* represents the soil erodibility factor; *K* represents the soil loss per unit area formed by unit rainfall erosion force under the standard plot; *L* is the slope length factor (dimensionless); *S* is the slope factor (dimensionless); *C* is the vegetation cover factor (dimensionless); and *P* is the soil and water conservation measure factor.

#### 2.3.4. Carbon Sequestration

Carbon sequestration services are mainly the function of green plants that absorb carbon dioxide (CO_2_) through photosynthesis, convert it into carbohydrates such as glucose, and fix it as organic carbon in the plant or the soil. The carbon sequestration function is important for regulating climate maintenance and balancing the stability of CO_2_ in the atmosphere, mitigating the greenhouse effect, and improving the living environment [60]. The amount of carbon sequestration was chosen as the evaluation index of the carbon sequestration function for ecosystems in the study [60].

(5) $$NEP=NPP-R_{h}$$

(6) $$R_{h}=0.592\times R_{s}^{0.714}$$

(7) $$R_{s}=1.55e^{0.031\times T}\times \frac{P}{P+0.68}\times \frac{0.58\times BD\times H\times (1-\delta /100)/10}{0.58\times BD\times H\times (1-\delta /100)/10+2.23}$$

where *NEP* is the ecosystem carbon sequestration (g C m^−2^ yr^−1^); *NPP* is the ecosystems’ net primary productivity (g C m^−2^ yr^−1^); and *Rs* is the carbon consumption by soil respiration (g C m^−2^ yr^−1^). where *T* is the mean annual air temperature, *P* is the annual precipitation, *BD* is the bulk density (g cm^-3^), *H* is the soil thickness (20 cm), and *δ* is the <2 mm fraction(%) of soil.

#### 2.3.5. Oxygen Release

Ecosystems’ oxygen release function refers to green plants’ function of absorbing carbon dioxide (CO_2_) from the atmosphere through photosynthesis, converting it into carbohydrates such as glucose, and releasing oxygen (O_2_). The oxygen release function of ecosystems is important for human society and the global climate balance. Studies have chosen oxygen release as an evaluation index of ecosystems’ oxygen release function [35].

(8) $$Q_{O_{2}}=NPP\times 1.19$$

where *Q*_*O*_2__ is the ecosystem oxygen release (g m^−2^ yr^−1^) and *NPP* is the ecosystem net primary productivity (g m^−2^ yr^−1^).

#### 2.3.6. Importance Analysis

In this paper, we use random and put-back sampling for the training set when constructing each decision tree, and 4/5 of the samples were used as the training set. The remaining 1/5 was used for accuracy validation, which is called Out-Of-Bag data. The misspecification rate of OOB is defined as the ratio of the number of misspecification to the total number of samples, while the OOB misspecification rate is used to evaluate the importance of the feature variables. In this paper, the replacement method is used to calculate the false positive rate of the OOB.

## 3. Results

### 3.1. Optimal Analysis Granularity of the Ecosystem Pattern

**Figure 2:**
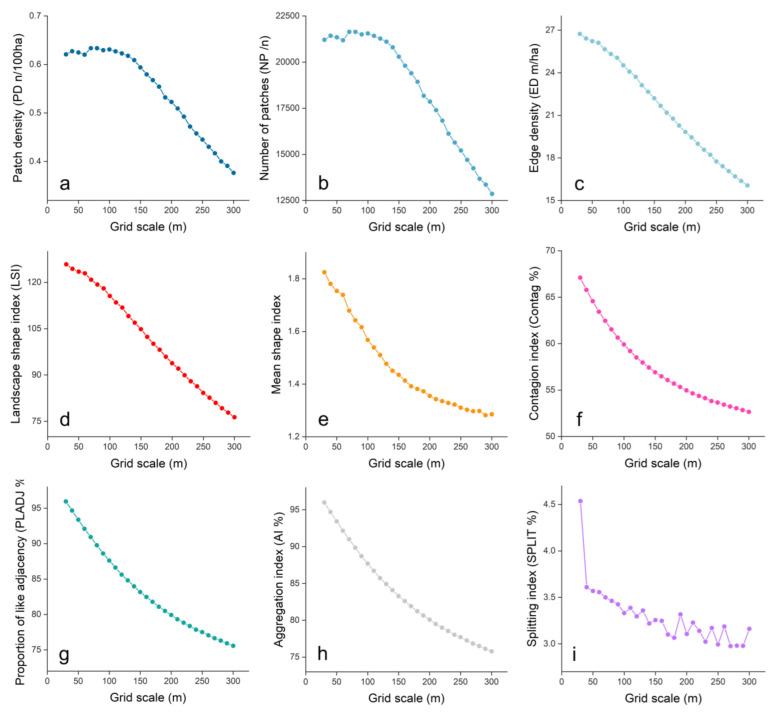
Change trend of the sensitive landscape index with granularity at the landscape level on Hainan Island. (**a**) Change trend of PD with granularity. (**b**) Change trend of NP with granularity. (**c**) Change trend of ED with granularity. (**d**) Change trend of LSI with granularity. (**e**) Change trend of SHAPE-MN with granularity. (**f**) Change trend of CONTAG with granularity. (**g**) Change trend of PLADJ with granularity. (**h**) Change trend of AI with granularity. (**i**) Change trend of SPLIT with granularity.

In this study, the sensitivity of each landscape index to spatial granularity was judged according to the coefficient of variation, and the landscape index with a coefficient of variation greater than 5% was set as the sensitive index. NP, PD, ED, LSI, SHAPE-MN, CONTAG, PLADJ, SPLIT and AI were sensitive indexes at the landscape level. In the seven years, the coefficient of variation of each landscape index in 1995 was higher as a whole, so the data from 1995 were selected for the trend analysis of the best spatial analysis granularity in the study area (Figure 2). Except for the SPLIT index, the other indexes showed a downward trend. The first inflection point of SPLIT appears at 110 m, indicating that the granularity has a great impact on SPLIT. Therefore, we chose 110 m as the optimal scale for the study of ecosystem services on Hainan Island.

### 3.2. Change in Ecosystem Type

**Figure 3:**
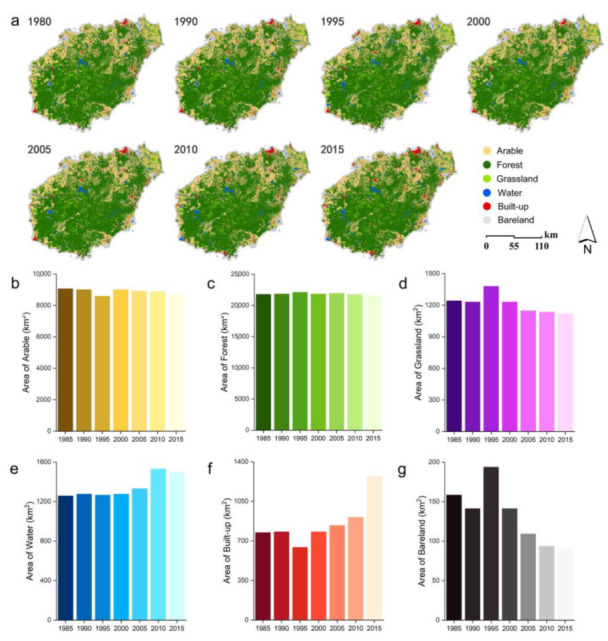
Distribution and long time series area of ecosystem types on Hainan Island from 1980 to 2015. (**a**) Temporal and spatial distribution of ecosystem types on Hainan Island from 1980 to 2015. (**b**) Change in arable area on Hainan Island from 1980 to 2015. (**c**) Change in forest area on Hainan Island from 1980 to 2015. (**d**) Change in grassland area on Hainan Island from 1980 to 2015. (**e**) Change in water area on Hainan Island from 1980 to 2015. (**f**) Change in built-up area on Hainan Island from 1980 to 2015. (**g**) Change in bareland area on Hainan Island from 1980 to 2015.

**Figure 4:**
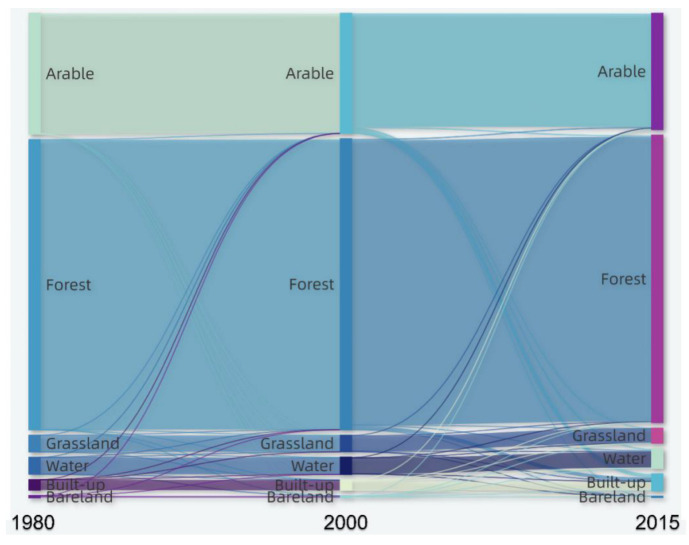
Distribution and long time series area of ecosystem types on Hainan Island from 1980 to 2015.

Hainan Island’s ecosystem type was mainly forest, with an area of 21,577.95 km^2^ in 2015, accounting for 62.9% of the province’s total area, distributed in the periphery of the built-up areas of its cities and counties, and mainly concentrated in its central mountainous areas. Next is arable land, an area of approximately 8722.7 km^2^, accounting for 25.5% of its total area, mainly consisting of paddy fields and distributed in its northern part’s flat terrain area. Built-up was approximately 1273.53 km^2^, accounting for 3.7% of its total area, forming urban centres in its coastal areas. The grassland and water areas were small and scattered (Figure 3a). In terms of ecosystem type changes, from 1980 to 2015, arable land (Figure 3b), forest (Figure 3c), and grassland (Figure 3d) decreased slightly, with decrease ratios of 0.98%, 0.55%, and 0.36%, respectively (Figure 4). Built-up (Figure 3e) and water (Figure 3f) demonstrated an increasing trend, with the increase ratio reaching 1.46% and 0.51%, respectively (Figure 4). The area of Hainan Island with no change in ecosystem type conversion during 1980–2015 accounted for 4.16% (Figure 4). This conversion mainly occurred in its coastal urban built-up areas, mainly urban expansion, with multiple changes manifested in its conversion of arable land and forest to built-up land (Figure 4). This increase in water was mainly due to the recent growth of the coastal mudflat areas.

### 3.3. Spatial and Temporal Characteristics of Ecosystem Services

**Figure 5:**
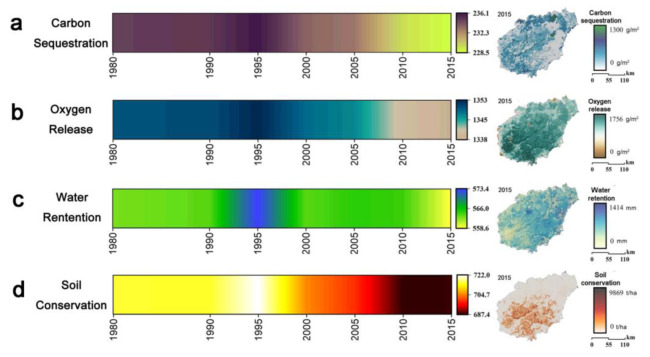
Time series heatmap of ecosystem services on Hainan Island from 1980 to 2015. (**a**) Time series heatmap of carbon sequestration on Hainan Island from 1980 to 2015. (**b**) Time series heatmap of oxygen release on Hainan Island from 1980 to 2015. (**c**) Time series heatmap of water retention on Hainan Island from 1980 to 2015. (**d**) Time series heatmap of soil conservation on Hainan Island from 1980 to 2015.

The functional quantities of Hainan Island’s main ecosystem services from 1980 to 2015 were obtained by the ecological model (Figure 5), and annual statistical data are displayed in Table 2. Hainan Island’s water content changed from 23.31 billion m3 in 1980 to 23.15 billion m3 in 2015. Soil conservation changed from 2.89 × 109 t in 1980 to 2.79 × 109 t in 2015. The carbon sequestration service changed from 9.68 × 106 t in 1980 to 9.42 × 106 t in 2015. The oxygen release service changed from 56.05 × 106 t in 1980 to 55.53 × 106 t in 2015. Figure 5 indicates that except for the central mountainous areas, the water retention of lakes, rivers, and reservoirs is high. A typical area is Songtao Reservoir, which is also the red line area designated for water source protection and water retention-related ecological protection. The areas with the highest soil conservation are concentrated in Baisha, Qiongzhong, Wuzhishan, and Baoting in the central area. While carbon sequestration generally had a high distribution in the northwest and central areas and a low distribution in the southeast area, higher oxygen release services were in the central and southern mountainous areas. In the past 35 years, Hainan Island’s major ecosystem services have declined (Figure 5), but the overall change is relatively small.

**Figure 6:**
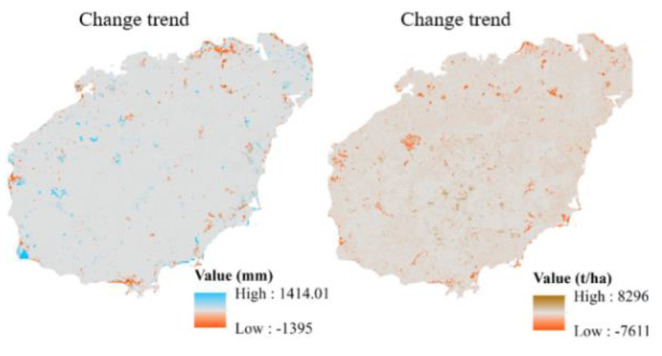

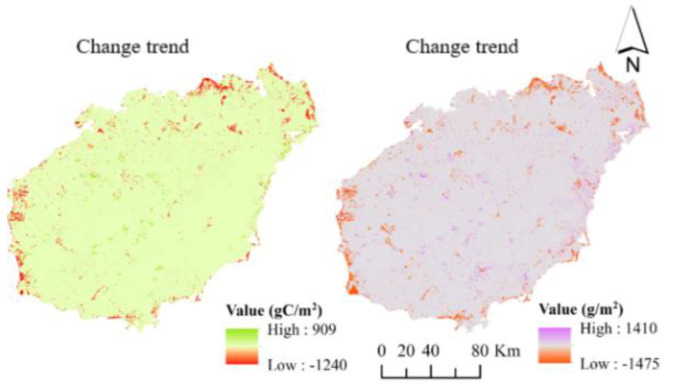
Trend distribution maps of average ecosystem services on Hainan Island from 1980 to 2015.

Figure 6 displays the trend change information of water retention, soil conservation, carbon sequestration, and oxygen release service. Further analysis of the increase and decrease in ecosystem services found that the decrease in water retention was mainly concentrated in the western, southwestern, eastern and northeastern parts of Hainan Island. The degradation of soil conservation resembles the decrease in water retention. The decrease in carbon sequestration was concentrated in the western, southwestern and northeastern areas of Hainan Island, such as western Dongfang, northern Haikou, southwestern Ledong, southern Sanya and Wenchang. Second, the declining areas of oxygen release service were mainly concentrated in the western, southwestern and northeastern areas of Hainan Island, such as Ledong, Dongfang, northern Haikou, eastern Wenchang and southwestern Sanya, while the rising areas were the central and northern areas of Hainan Island, such as Baisha, Danzhou, Wuzhishan, Baoting, and Qiongzhong. The declining areas of oxygen release service were mainly concentrated in the western, southwestern and northeastern areas of Hainan Island, such as Ledong, Dongfang, northern Haikou, eastern Wenchang, and southwestern Sanya, while the rising areas were the central and northern areas of Hainan Island, such as Baisha, Danzhou, Qionghai, and Baoting. In general, the declining areas are concentrated in the urban expansion areas, mainly in coastal areas, while the amount of ecosystem service function in the central dispersed areas has increased.

### 3.4. Drive Analysis

#### 3.4.1. Drive Analysis of Human Activities

To further analyse the negative drivers of human disturbance of urban expansion and the positive drivers of ecological control in the central mountainous region, this paper extracted the ecosystem service time series within the scope of human disturbance and within the scope of nature reserves using Hainan Island’s 2015 construction land boundary as well as the boundaries of its five typical national nature reserves and compared them with the island-wide mean ecosystem service time series. This comparison was made with the island-wide ecosystem service time series.

**Figure 7:**
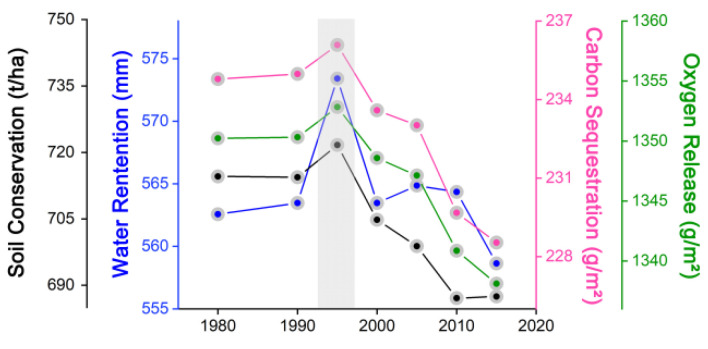
Time series diagram of ecosystem services in Hainan Island between 1980 and 2015.

**Figure 8:**
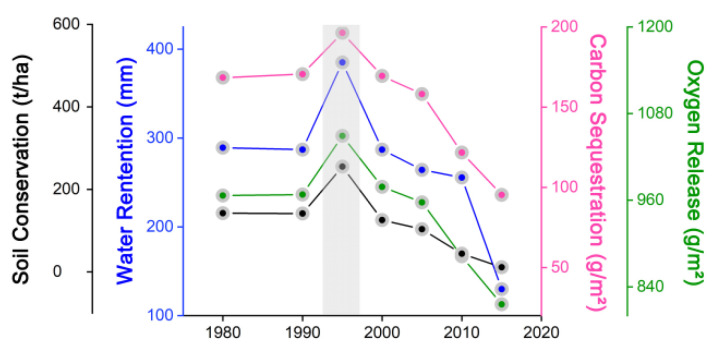
Time series diagram of ecosystem services in urban areas in Hainan Island between 1980 and 2015.

Figure 7 and Figure 8 display the time series diagram of Hainan Island’s ecosystem services and urban areas, respectively, which demonstrated that both ecosystem services have consistent trends, with all four variables indicating major peaks in 1995 and an overall decreasing trend, with Pearson correlation coefficients of 0.88 (water conservation), 0.93 (soil conservation), 0.98 (carbon sequestration services), and 0.96 (oxygen release services), further indicating that urban sprawl has been the dominant factor in the decline of Hainan Island’s ecosystem services in the last 35 years.

**Figure 9:**
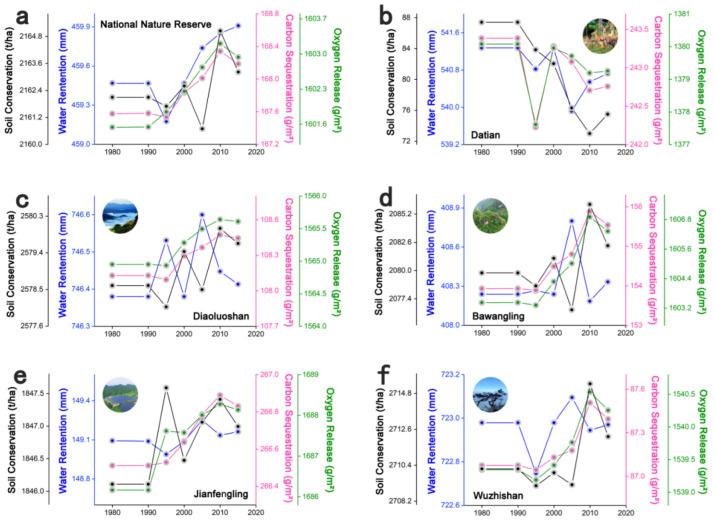
Broken line diagram of the ecosystem service time series of national nature reserves from 1980 to 2015. (**a**) Broken line diagram of the ecosystem service time series of all national nature reserves from 1980 to 2015. (**b**) Broken line diagram of the ecosystem service time series of Datian national nature reserves from 1980 to 2015. (**c**) Broken line diagram of the ecosystem service time series of Diaoluoshan national nature reserves from 1980 to 2015. (**d**) Broken line diagram of the ecosystem service time series of Bawangling national nature reserves from 1980 to 2015. (**e**) Broken line diagram of the ecosystem service time series of Jianfengling national nature reserves from 1980 to 2015. (**f**) Broken line diagram of the ecosystem service time series of Wuzhishan national nature reserves from 1980 to 2015.

The time-series changes in ecosystem services in five typical national nature reserves on central Hainan Island are depicted in Figure 9, demonstrating that the overall ecosystem services of national nature reserves underwent an increasing trend from 1980 to 2015. Among them, except for the Dada National Nature Reserve, the ecosystem services of the remaining four national nature reserves increased, which tends to indicate that Hainan Island’s ecological services have declined in the last 35 years due to urban expansion. However, the ecosystem services in important nature reserves were increasing, indicating that conservation and management, such as ecological restoration and rehabilitation, have been more effective.

#### 3.4.2. Drive Analysis of Natural Factors

**Figure 10:**
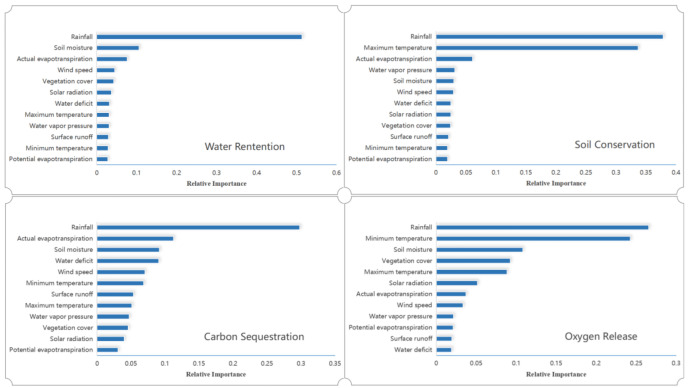
Relative importance ranking of natural factors for the spatial distribution of ecosystem services on Hainan Island.

Ecosystem service functions depend on ecosystem structure and processes in a certain time and space. Moreover, environmental factors combine to influence ecosystem structural composition and distribution patterns. This study further investigated the drivers and contributions of natural environmental factors to the spatial partitioning of ecosystem services by using TerraClimate reanalysis data based on the random forest algorithm [61,62] Based on the comprehensiveness and accessibility of the indicators, we selected 12 natural environment indicators: AET, DEF, PET, RO, SOIL, SRAD, TMN, TMX, VPD, VS, PRE and NDVI. Figure 10 illustrates the ranking (in descending order) of the spatially distributed drivers of water content, soil conservation, carbon sequestration, and oxygen release. The three most important environmental drivers for water harvesting were rainfall, soil moisture, and actual evapotranspiration. The three most important environmental drivers for soil conservation were rainfall, maximum temperature, and actual evapotranspiration. The main environmental drivers for the carbon sequestration service were rainfall, actual evapotranspiration, and soil moisture. The main environmental drivers for oxygen release service were rainfall, minimum temperature, and soil moisture. Among these, rainfall indicators were much more important for water content function than other ecosystem service functions. Overall, the natural environmental drivers of the spatial distribution of Hainan Island’s four ecosystem services were mainly rainfall, soil moisture, actual evapotranspiration, maximum temperature and minimum temperature, while the relative importance of potential evapotranspiration was relatively low.

## 4. Discussions

### 4.1. Comparison of Ecosystem Services with Previous Estimates

This paper quantified four significant ecosystem services identified on Hainan Island between 1980 and 2015. The characteristics of spatial-temporal patterns and driving forces of ecosystem services were explored. The results showed that there was an improvement in the year 1995, but the figures for 2015 indicated a slight decline when compared to the figures for 1980. Although a relative decline occurred, the amounts per unit area of water retention, soil conservation, carbon sequestration, and oxygen release services on Hainan Island were higher than those of most other regions in China, when compared to previous studies.

Wu et al. [63] and Xiao and Ouyang [64] estimated water retention services at the national-scale in China, which indicated that Hainan Island was one of the high regions of water retention capacity and had an increasing trend from 2000 to 2014. The increasing trend was in accordance with our study during this period. In contrast, the amount of water retention services in our study demonstrated a decrease in the year 2015. Chen et al. [65] reported that the spatial patterns of soil conservation in China were obviously different from 1970 to 2009 and the highest soil rate appeared in southeastern China including Hainan Island and other provinces. Our estimation was almost the same service capacity as the result of Chen et al. [65]. Tang et al. [66] estimated the carbon stock in China’s forests, shrublands, grasslands and croplands based on field campaigns. They showed that the total carbon stock of these terrestrial ecosystems amounts to 79.24 Pg C. Due to a large area of tropical rainforest with high sequestration potential in the central part of Hainan Island, it was defined as an important carbon sequestration area in China, which is regarded as a priority conservation area with the principle of ‘smaller areas receive larger ecosystem services’.

Previous studies have mostly explored changes in single ecosystem services from a nationwide perspective. Although Hainan Island is an area with high ecosystem service density obviously, it has not attracted much attention from researchers. The spatial distribution and temporal variations in Hainan Island are not well understood. We analysed how ecosystem type transformation affected ecosystem services from spatial-temporal processing.

### 4.2. Driving Forces of Ecosystem Services

Our results indicated that ecosystem services in nature reserves have had an increasing trend during these decades. However, in urban cities located in coastal areas, ecosystem services have obviously declined. Because of large protected areas and ecological restoration projects, they have contributed to an important increase in ecosystem services. Therefore, there was no significant downward trend for the whole island. The persistent increase in ecosystem services depends on management strategies and the extent of human activities [67].

Rainfall is generally the determinant factor for ecosystem systems, which has been demonstrated by previous studies [64,65,66]. In our study, 12 natural factors were evaluated to identify the relative importance related to ecosystem systems. We also found that rainfall was the most significant factor among them. In contrast, the second driving factors were different in each kind of ecosystem service. Soil moisture, maximum temperature, actual evaporation and minimum temperature are the second driving factors for water retention, soil conservation, carbon sequestration and oxygen release, respectively. Compared with human impacts, natural factors are likely to have more impacts on ecosystem services.

### 4.3. Limitations

This study analysed the spatial and temporal dynamics and drivers of ecosystem services on Hainan Island in the last 35a in both temporal and spatial dimensions. However, this study also has some shortcomings. First, the analysis of ecosystem service drivers in this study was only explored from empirical models, but not from physical models such as land-air feedback. In addition, only four ecosystem services were selected to measure the ecosystem service function of Hainan Island, which has a certain impact on the scientific reference value of this study. Then, due to objective factors, the research time point of this study was only up to 2015, which has some impact on the scientific reference value of this study. Finally, as the highest spatial resolution reanalysis data are available, the Terraclimate dataset is still inadequate when applied to Hainan Island, which has an area of only 35,400 km^2^. Therefore, we will conduct further research on the analysis of ecosystem services and their drivers on Hainan Island in the future around the features of high timeliness, high accuracy and deep level.

## 5. Conclusions

In the context of rapid urbanization and drastic climate change, the changes in Hainan Island’s ecosystem services have an important role in maintaining China’s stable biodiversity and balanced ecosystems. Therefore, this study combines multisource remote sensing data as well as reanalysis data to analyse the spatial and temporal characteristics of ecosystem services and their drivers on Hainan Island from 1980–2015, with the following main conclusions.

(1) Hainan Island’s ecosystem type was mainly forest, accounting for 62.9% of its total area, and was mainly concentrated in its central mountainous region. Arable land accounted for 25.5% of its total area and was mainly paddy land, mainly distributed in the flat terrain of its northern part. Built-up accounts for 3.7% of its total area, forming a clear urban agglomeration centre in its coastal areas. For nearly 35 years, Hainan Island’s overall ecosystem type conversion was not dramatic, and this area accounted for a relatively small proportion.

(2) The amount of the four types of ecosystem services assessed (water retention, soil conservation, carbon sequestration services, oxygen release services) from 1980–1995 demonstrated an upwards trend and thereafter indicated a downward trend. In terms of spatial distribution, the declining areas were mainly concentrated in urban expansion areas, while the amount of ecosystem service function in the central, dispersed, and important ecological function areas demonstrated an increase.

(3) Urban expansion and ecological control have negatively and positively impacted ecosystem services. Hainan Island’s urban built-up area during 2015 was extracted to compare its ecosystem services with the multiyear change trend of its overall ecosystem services, and the two changes were highly correlated. Five of Hainan Island’s typical national nature reserves were extracted to compare their changes in ecosystem services, and the region had an upwards trend. This is an indication that the regional ecology’s conservation and control can mitigate the impact of the declining amount of ecosystem services resulting from urban expansion and other changes.

## Figures and Tables

**Table 1:** Detailed description of the data.

Data Name	Spatial Resolution	Time Resolution	Source	Note
CLUD	30 m	1980–2015	RESDC. ^a^	Calculate ecosystem services
Sunshine hours	/	1980–2015	CMSDSSN. ^b^	Calculate carbon sequestration
Soil texture	/	/	WSD. ^c^	Calculate carbon sequestration
soil organic	/	/	WSD. ^c^	Calculate carbon sequestration
soil capacity	/	/	WSD. ^c^	Calculate carbon sequestration
National nature reserve	/	/	RESDC. ^a^	Boundary data
Base pond area	/	/	WRDS. ^d^	Calculate water retention
AET	4638 m	Monthly	NCAR ^e^	Sensitivity analysis
DEF	4638 m	Monthly	NCAR ^e^	Eco-environmental benefits analysis
PET	4638 m	Monthly	NCAR ^e^	Delineate LUBs
RO	4638 m	Monthly	NCAR ^e^	Driven analysis at spatial scale
SOIL	4638 m	Monthly	NCAR ^e^	Driven analysis at spatial scale
SRAD	4638 m	Monthly	NCAR ^e^	Driven analysis at spatial scale
TMN	4638 m	Monthly	NCAR ^e^	Driven analysis at spatial scale
TMX	4638 m	Monthly	NCAR ^e^	Driven analysis at spatial scale
VPD	4638 m	Monthly	NCAR ^e^	Driven analysis at spatial scale
VS	4638 m	Monthly	NCAR ^e^	Driven analysis at spatial scale
PRE	4638 m	Monthly	NCAR ^e^	Driven analysis at spatial scale
Landsat 5/8	30 m	16-day	NASA. ^f^	Calculate NDVI

Note: ^a^ Resource and Environmental Science and Data Center (https://www.resdc.cn/, accessed on 5 May 2022). ^b^ China Meteorological Science Data Sharing Service Network (http://data.cma.cn/, accessed on 5 May 2022). ^c^ World Soil Database (https://iiasa.ac.at/models-and-data/harmonized-world-soil-database, accessed on 5 May 2022). ^d^ Water Resources Department (http://swt.hainan.gov.cn/, accessed on 5 May 2022). ^e^ National Center for Atmospheric Research (https://climatedataguide.ucar.edu/, accessed on 5 May 2022) [45]. ^f^ National Aeronautics and Space Administration (https://www.nasa.gov/, accessed on 5 May 2022).

**Table 2:** Changes in the physical quantities of ecosystem services on Hainan Island from 1980 to 2015.

Type	1980	1990	1995	2000	2005	2010	2015	1980 to 2015
Water retention (10^9^ m^3^)	23.31	23.34	23.76	23.34	23.40	23.39	23.15	−0.16
Soil conservation (10^9^ t)	2.90	2.90	2.93	2.86	2.84	2.79	2.79	−0.11
Carbon sequestration (10^6^ t)	9.68	9.69	9.73	9.63	9.61	9.47	9.42	−0.26
Oxygen release (10^6^ t)	56.05	56.06	56.16	55.98	55.92	55.64	55.53	−0.52

## Data Availability

Not applicable.
